# Imaging strategies for vesicoureteral reflux diagnosis

**DOI:** 10.1007/s00467-006-0396-8

**Published:** 2007-07-01

**Authors:** Constantinos J. Stefanidis, Ekaterini Siomou

**Affiliations:** 1 Department of Nephrology, “P. & A. Kyriakou” Children’s Hospital of Athens, Goudi, 14562 Athens, Greece; 2 Department of Pediatrics, University Hospital of Ioannina, Ioannina, Greece

**Keywords:** Urinary tract infection, Acute pyelonephritis, Vesicoureteral reflux, Reflux nephropathy, Diagnostic imaging

## Abstract

The prevalence of vesicoureteral reflux (VUR), although reported to be low in the general population, is high in children with urinary tract infection (UTI), first degree relatives of patients with known VUR and children with antenatal hydronephrosis. In addition, it has been shown that VUR and UTIs are associated with renal scarring, predisposing to serious long-term complications, i.e., hypertension, chronic renal insufficiency and complications of pregnancy. Therefore, diagnostic imaging for the detection of VUR in the high-risk groups of children has been a standard practice. However, none of these associations has been validated with controlled studies, and recently the value of identifying VUR after a symptomatic UTI has been questioned. In addition, several studies have shown that renal damage may occur in the absence of VUR. On the other hand, some patients, mainly males, may have primary renal damage, associated with high-grade VUR, without UTI. Recently, increasing skepticism has been noted concerning how and for whom it is important to investigate for VUR. It has been suggested that the absence of renal lesions after the first UTI in children may rule out VUR of clinical significance and reinforces the redundancy of invasive diagnostic techniques. Therefore, the priority of imaging strategies should focus on early identification of renal lesions to prevent further deterioration.

## Introduction

Urinary tract infection (UTI) is relatively common in young children and occurs in 2.2% for boys and 2.1% for girls younger than 2 years [1]. The prevalence of UTI in girls increases in older children, and it has been estimated that 8.4% of girls and 1.7% of boys will develop at least one UTI by the age of 7 years [2]. It has been long recognized that UTI is a marker for abnormalities of the urinary tract, the most common problem being primary vesicoureteral reflux (VUR). The prevalence of VUR is 30% to 40% in children with UTI and appears to decrease with age [3]. It is also diagnosed in 9% to 11% of neonates with antenatal hydronephrosis [4, 5], in 32% of siblings [6] and in 66% of offspring of known VUR patients [7]. In contrast, the incidence of VUR in healthy children was less than 2% in studies conducted between the 1950s and the 1970s [8]. This association led to the concept that VUR played a significant role in the pathogenesis of UTIs, acute pyelonephritis (APN) and renal scarring and has been the basis for diagnostic procedures [9]. However, none of these associations have been validated with controlled studies. In addition, a significantly higher prevalence of VUR in normal population has been calculated from epidemiological data of children without UTI [10]. If these doubts are correct, it would argue against the clinical significance of VUR and the routine use of voiding cystourethrography (VCUG).

## Considerations for planning the investigation for VUR

Coarse renal scarring of one or both kidneys is associated with VUR and UTI and is called reflux nephropathy (RN) [11]. Fortunately, RN is much less common than VUR and UTI, although potentially more serious. In the past, children with RN were considered to be at risk for recurrent infections and long-term problems, such as hypertension, chronic renal insufficiency (CRI) and complications of pregnancy [12]. However, no distinction was made between children with UTI, VUR and primary scars and UTI, VUR and secondary scars. This distinction is very important, because CRI occurs in the first group and is almost never seen in the second (at least in childhood). The main role of diagnostic imaging in UTI is to identify the children with a high risk for developing RN. Increasing skepticism has been noted recently concerning when and for whom it is important to investigate for VUR. This is mainly the result of a systematic review of recent publications made based on patients with RN that revealed the following information.

### Primary and secondary reflux nephropathy

Data from studies in newborns with VUR detected in the investigation of fetal hydronephrosis documented the presence of congenital renal damage in the absence of UTI [4, 5, 13]. Actually, most of the severely affected kidneys had no exposure to UTI (85%) [13]. Their primary renal damage is usually associated with higher grades of VUR [14], and both findings may be the result of embryonal abnormality of the ureteral bud. These patients with congenital anomalies of the kidney and urinary tract (CAKUT) are mainly boys with global parenchymal reduction, i.e., small kidneys without focal scars. Renal hypo-dysplasia is characterized by a reduction in nephron number, a small overall kidney size and/or disturbed organization of the renal tissue with lack of corticomedullary differentiation and the presence of cysts that, on occasion, massively distend the organ [15]. In a recent study, mutations or variants in five important renal developmental genes that are associated with syndromal renal malformations were detected in 17% of children who presented with renal hypo-dysplasia and CRI [16]. This percentage is expected to increase in the future, when mutation analysis of genes for which renal maldevelopment has been demonstrated in genetically modified animal models will be performed.

**Fig. 1:**
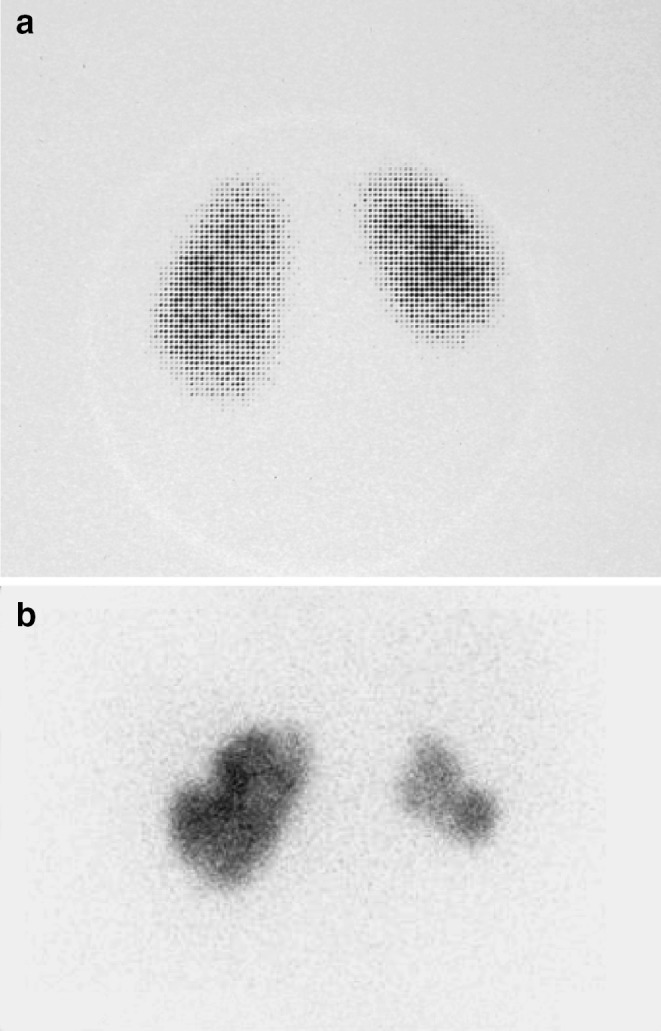
**a** DMSA scan of a 2-year-old girl with persistent, bilateral grade II VUR and dysfunctional voiding 6 months after the first documented febrile UTI. A smaller size of right kidney is demonstrated, compared to the left kidney with focal and generalized reduction in radiotracer uptake in the poles and indentation of the renal contour. The left kidney also presents a lack of homogeneity in DMSA uptake, mainly in the lower pole. **b** A DMSA scan 9 years later, after stopping the follow-up and antibiotic prophylaxis on the family’s own initiative and after breakthrough UTIs. The right kidney demonstrates further reduction of the size, and new scars are seen in both kidneys

Children with primary renal damage should be distinguished from the secondary RN, which is the result of recurrent febrile UTIs and occurs mainly in girls of older age. Children with secondary RN have normal kidneys initially, and segmental scarring is usually diagnosed after infancy. Recurrent UTI is the most important pathogenetic factor for acquired renal scarring [12] (Fig. 1). Although the presence of moderate to severe VUR remains a significant risk factor for renal scarring [17], often renal scarring is associated with no or low-grade VUR [18, 19]. Unfortunately, it is not always possible to distinguish primary and secondary lesions with a renal cortical scintigraphy with technetium 99m dimercaptosuccinic acid (DMSA) scan. There is evidence that some renal scars associated with high-grade VUR that have been considered to be secondary may actually represent primary fetal nephropathy [20]. Therefore, acquired RN can only be documented if a DMSA has been done before and after an APN.

### Bladder dysfunction and VUR

Several studies have reported a high frequency of bladder dysfunction (bladder instability and detrusor-sphincter dyscoordination) in children with VUR [21]. Children with bladder instability are characterized by sphincter constrictions during bladder filling in order to maintain continence during a contraction of the bladder. These patients have symptoms of incontinence, urgency and frequency. They usually have low or no residual volume. In contrast, the characteristic of children with dyscoordinated bladder is obstruction during voiding. This is a much more serious problem; however, recently milder forms have been described. These children usually have urgency, diurnal and nocturnal incontinence, interrupted voiding and frequent UTIs [21]. They also often have high bladder capacity (greater than twice the expected normal value for age) and high residual urine (greater than 20% of bladder capacity) [22].

Bladder dysfunction is frequently unrecognized and is associated with delayed VUR resolution and an increased rate of breakthrough UTI [23]. It is of interest that after the disappearance of VUR, UTIs occurred in 26% of children with dysfunctional voiding. It was suggested that the appropriate evaluation and management of this problem should be an integral part of the treatment of every child with VUR [23]. It was also recently proposed that VUR is a heterogeneous disease and in some patients should be regarded as a marker of a combination of disorders that include primary renal lesions, altered urinary bladder function and a predisposition to UTI [9].

### VUR as a predictor of renal damage

APN may occur in the absence of demonstrable VUR [24], and once it has occurred, ultimately renal scarring is independent of the presence or absence of VUR [25, 20]. Actually, in a recent multicenter study permanent renal damage was diagnosed in 52% of children without VUR [26]. The investigation of these patients with a DMSA scan, despite the negative findings on the VCUG, may possibly lead to the prevention of long-term complications. These children may have primary renal damage with no VUR, or VUR could have spontaneously resolved before the UTI that prompted the imaging studies. Another possibility is that some bacteria access renal parenchyma from the urinary bladder either by direct ascent or by hematogenous spread [27]. Finally under-diagnosis of VUR by VCUG is well documented in studies with radionuclide cystography (RNC) [17].

In a recent meta-analysis it was found that a positive VCUG increased the risk of renal damage by about 20%, whereas a negative VCUG increased the chance of no renal involvement by just 8% [27]. In addition, an abnormal DMSA was found only in 16% of children with VUR aged less than 1 year with a normal renal ultrasound (US), and 50% of scarred kidneys did not have associated VUR [28]. It is also documented that a normal DMSA scan after the first UTI can exclude VUR of clinical significance irrespective of age and infection characteristics [29]. It is of interest that after 1 year of follow-up monitoring, mild/moderate VUR does not increase the incidence of UTI, APN or renal scarring after APN [30]. However, in another recent study it was found that 40% of children with VUR had persistent renal parenchymal defects on a DMSA scan performed 6 months after the last febrile UTI. In contrast, renal lesions were found in only 6% of subjects without VUR with at least two febrile UTIs or one febrile UTI whose antibacterial treatment was started more than 4 days after the onset of fever. The higher prevalence of persistent renal defects in this study was explained as the result of the higher age of the patients (50% of them were over 2 years of age) with the possible accumulation of scars from previous APN [17].

It has been long recognized that the chance of developing RN is much higher in younger children, with babies at greatest risk [31]. In addition, children with normal US and DMSA scan after a UTI have a negligible risk of developing a scar after their 4th birthday [32]. A possible explanation is that most vulnerable subjects have already had their kidneys scarred in infancy, and children reaching 4 years without a scar are at minimal risk of scarring in the future [33]. This may be because they never had VUR, or they have VUR, but no compound papillae and intrarenal reflux. In addition, there is evidence that maturation does not lead to an increased resistance to scarring after a UTI. It was demonstrated that adult pigs are as vulnerable to scarring as piglets [34], and there have been reports of typical reflux nephropathy lesions appearing in transplanted mature human kidneys, both histologically and on DMSA scanning [35]. It was also found in a prospective study that the risk of renal scars after APN does not diminish with age [36]. Therefore, it should be recognized that progressive RN might occur in older children with VUR and UTI, who had abnormal findings in their initial DMSA scans.

### Outcome of children with VUR and RN

Our understanding of the natural history of VUR was based on old long-term follow-up studies that detected renal scars with intravenous urography (IVU). Renal scars took months or years after an APN to develop to a size that could be readily detected with IVU. In recent studies, IVU has been replaced by DMSA, which is significantly more sensitive to detect focal kidney abnormalities. Obviously, the long-term outcome of these children might not be relevant to patients with lesions detected by IVU [37]. Changes on the DMSA scan were found in 86% of 76 children during APN. However, 27% of the lesions resolved within 2 months after the APN and 49% within 2 years [38]. Other sequential studies, evaluating patients with an initially abnormal DMSA scan, revealed that renal defects persisted as renal scarring in 36%–52% of kidneys [20]. In addition, new renal scarring developed in 25% of kidneys with VUR compared with 37% without VUR [25]. Therefore, once APN has occurred, ultimately renal scarring is independent of the presence or absence of VUR.

In a recent retrospective cohort study, it was found that after a long-term follow-up 2.8% of children with VUR had a GFR <75 ml/min per 1.73 m^2^ in two consecutive examinations, and the prevalence of hypertension was 2.7% [39]. There was no difference in progression to chronic renal insufficiency between boys (3.8%) and girls (2.4%). Although boys had a more severe pattern at baseline, girls had a greater risk of dysfunctional voiding and recurrent UTI during follow-up. The decline of renal function occurred in those patients with severe bilateral renal damage or in those children with contralateral primary renal damage. The majority of these lesions are primarily determined, and, probably, the appropriate management of primary VUR could scarcely contribute to improving the prognosis of these patients [39]. Data from various registries have shown that between 5% and 12% of patients entering end-stage renal disease programs have RN. However, the total number of these patients is a small percentage of all patients with RN. Therefore, only a very small percentage of children with VUR and RN are at risk to develop end-stage renal disease in adulthood [12]. In addition, estimates of undesirable outcomes of RN in adulthood, such as hypertension and end-stage renal disease, are based on the mathematical product of probabilities at several steps, each of which is subject to bias and error.

## Imaging techniques

**Fig. 2:**
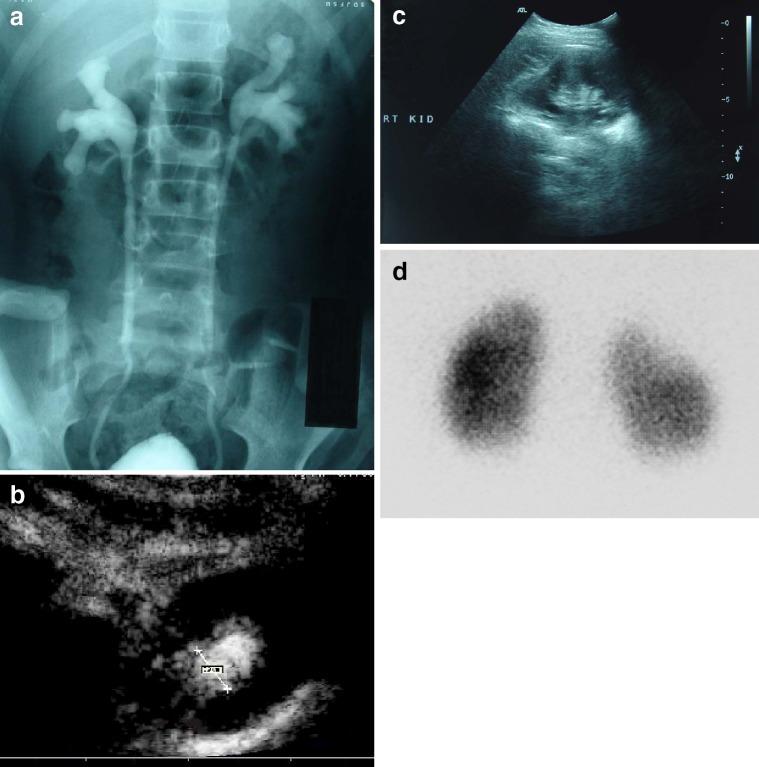
Radiological imaging of a 7-year-old girl with a history of acute pyelonephritis. **a** Voiding cystourethrography shows bilateral vesicoureteral reflux (VUR) grade III. **b** Contrast-enhanced harmonic voiding urosonography (VUS) (transverse section in prone position) shows also VUR grade III. The same image was found in VUS on the left kidney (not shown). **c** Ultrasound of the right kidney reveals irregularity of renal outline and focal thinning of renal cortex in the upper pole, findings compatible with scar. **d** Posterior view of 99mTc-dimercaptosuccinic acid (DMSA) scan 6 months after an acute pyelonephritis shows a focal defect in radiotracer uptake of the upper pole on the right kidney, indicating the presence of a renal scar

There has been an ongoing debate for several years about the most effective imaging strategy for children with UTI. Some centers focus mainly on detection of renal damage using US and/or DMSA. Other centers focus on the detection of VUR using VCUG or RNC or voiding urosonography (VUS). However, the majority of protocols recommend the complete functional and morphological evaluation of the child’s urinary tract. All these imaging techniques provide different information about the kidneys and urinary tract (Fig. 2). Therefore, integration of the data of the imaging results will provide the appropriate information for optimal management. In addition, the communication between radiologists and clinicians is fundamental for the integration of the imaging results with clinical and laboratory data leading to the optimal management.

### Urinary tract ultrasound and DMSA scan

US is a noninvasive technique with no ionizing radiation and is generally accepted as the primary imaging method for the evaluation of the kidneys and the bladder and the assessment of pelvic and calyceal dilatation, but it offers poor anatomical information about the urethra and the ureters [40]. However, US is neither sensitive, nor specific for detecting VUR [41]. It is of interest that 74% of refluxing renal units had normal US and 28% of them had VUR grade 3 or higher [42]. Indirect US signs, such as thickening of the renal pelvic, ureteric or bladder wall, as well as trabeculation or residual volume after voiding may increase the detection rate for VUR to as high as 85%, but still with a low specificity [40]. US findings, suggestive of renal scarring, may be a focal thinning of the renal cortex with or without indentation of the renal contour. In a systematic review by Roebuck et al., the sensitivity of US for scarring compared to DMSA ranged from 37% to 100%, and the specificity from 65% to 99% [43]. In another recent study, it was also found that the US had good specificity for the detection of renal scarring compared with DMSA. However, the sensitivity was low [44]. It is not clear that the additional sensitivity of DMSA translates directly to additional information of clinical importance. On the other hand, radiation exposure is an important limitation of DMSA (Table 1), especially when it is repeated several times. In addition, radionuclide studies are not as widely available for infants and young children as other modalities. Several pitfalls in the interpretation of the DMSA findings have been reported. Uncomplicated simple duplex kidneys may have greater uptake in the duplex kidneys or cause an erroneous impression of a small poorly developed contralateral kidney [45]. Similarly, a damaged duplex kidney may have normal relative function. Duplex systems are not always recognizable on DMSA scans. The relative function of each kidney is also normal in patients with bilateral renal damage. These pitfalls might be avoided when both a DMSA and an US are performed.

**Table 1:** Effective radiation dose of imaging techniques

Imaging	Effective dose (mSV)
Voiding cystourethrography	0.14–1.56 [48]
Direct radionuclide cystography	0.04–0.09 [92]
DMSA scan	1.10–1.18 [93]

In summary, US is an excellent modality for detecting structural renal abnormalities. However, the use of US in the detection of scarring remains controversial.

### Voiding cystourethrography

VUR has been traditionally detected by VCUG. Recently, digital and pulsed fluoroscopy have enabled a significant reduction of the radiation dose and reliable documentation of the findings [46–49]. VCUG provides images with fine anatomical detail, including the bladder and the urethra. The most important limitation of VCUG is the radiation exposure, particularly to the gonads [40, 50]. Furthermore, catheterization is painful and not entirely without the risk of iatrogenic infection. The incidence of post-VCUG infections was 6% to 22% in studies published in the 1970s. However, it was recently documented that with a prophylaxis protocol, a low incidence of symptomatic UTI (1.7%) was observed [51]. An alternative is to use suprapubic VCUG to avoid this complication. However, it was reported that suprapubic puncture took a little longer than urethral catheterization and scored slightly worse for discomfort. In addition, most families felt strongly that urethral catheterization seemed safer and preferable to suprapubic puncture [52].

Since VUR may be intermittent, the sensitivity in detecting VUR can be improved with cyclic procedures, i.e., filling the bladder and having the infant void around the catheter two or more times [53–55]. Cyclic procedures should only be used with RNC and VUS because of the unacceptably high radiation burden in cyclic VCUG [53].

A plain abdominal radiography, performed before the VCUG, may be helpful to provide some important information by detecting small calculi, spinal or sacral anomalies, bowel dilatation and stool retention [40].

### Radionuclide cystourethrography

There are two methods of RNC. The first, direct RNC, requires bladder catheterization and instillation of the radionuclide. Indirect RNC does not need bladder catheterization, but can only be performed in toilet-trained children, following a dynamic renogram, after intravenous injection of Tc-99m MAG3 or DTPA [41]. RNC provides less gonadal radiation (Table 1) and continuous monitoring, but the anatomic details are poor [40, 50].

With VCUG and direct RNC, both the filling and the micturition phase can be studied, whereas only the micturition phase may be studied with indirect RNC [56]. VUR was demonstrated only in the filling phase in some studies. Therefore, it was suggested that with indirect RNC a significant number of children with VUR will not be diagnosed [57]. In contrast, a high sensitivity of indirect RNC compared with direct RNC was documented by others [58], and it was recommended that those children >3 years of age who are toilet trained should undergo indirect RNC [59].

### Voiding urosonography

Contrast-enhanced voiding urosonography (VUS) with microbubbles containing contrast medium has comparable diagnostic accuracy to VCUG particularly for dilating VUR [60–63] and compared to direct RNC [64]. In addition, low-grade VUR has become reliably detectable with the use of contrast harmonic imaging [53, 65, 66]. Cyclic VUS detected 25% more VURs than the conventional (one cycle only) VUS [67]. Recently, a second cycle of contrast-enhanced harmonic VUS with no added additional dose of contrast medium has been reported to disclose significantly more cases of VUR at no additional cost for the examination [68]. The disadvantages of VUS are the less accurate grading and the poor anatomical information of the ureters and the urethra [40]. In addition, the longer examination time required for the VUS is an obstacle. However, exposure of the child to radiation is completely avoided, which is even more important in those children who must present for several follow-ups [63].

Comparisons among VUS, VCUG and RNC have shown a high concordance regarding the diagnosis of VUR. VUS was found to be more sensitive when compared to VCUG. Moreover, the refluxes missed by VCUG are predominantly of higher grade and thus clinically more important than those missed by VUS [69].

### Magnetic resonance urography (MRU)

MRU is a new technique for evaluating the urinary tract, and it is expected to replace traditional diagnostic methods, because it does not use ionizing radiation [40, 70]. Gadolinium-enhanced dynamic MRU allows better assessment of the urinary tract in neonates and infants than the US and IVU, with additional functional information [71]. MRU thus has the potential to replace IVU for many indications. In a recent study, post-gadolinium magnetic resonance imaging was compared with DMSA [72]. Sensitivity and specificity of magnetic resonance imaging in the detection of pyelonephritic lesions were found to be 91% and 89%, respectively, and there was no statistically significant difference in lesion detection between these modalities. Moreover, magnetic resonance imaging was superior in discriminating acute pyelonephritic lesions and permanent renal damage in early stages of disease [72]. MRU, with the use of optimal imaging sequences, correctly depicts anatomy and allows assessment of the urinary tract better than US and IVU, with additional angiographic and functional information. However, the use of MRU is presently restricted because it is expensive, not widely available, time-consuming and requires sedation of the young child [40]. MRU might become the imaging modality of the future, but our experience is quite limited today.

## Selection criteria for imaging methods in the diagnosis of VUR

The selection of an imaging method for VUR depends mainly on the patient population (age and gender). However, individual experience, the equipment available locally, as well as health care costs may influence the decision of the selected imaging method for the diagnosis of VUR. Many centers recommend VCUG as the first examination modality for VUR in boys, with a specific request for urethral and/or bladder imaging, and for severe antenatal hydronephrosis and abnormal kidney on renal US or DMSA scan [40, 50]. However, it is suggested that RNC might replace VCUG with the exception of male infants with gross bladder and/or bilateral pelviureteric dilatation by sonography, suggesting posterior urethral valves [73]. On the other hand, it is generally accepted that the primary indications for RNC or VUS are follow-up examinations and the screening of asymptomatic siblings of patients with VUR [40, 50, 69].

## Initial work-up in high risk groups

The prevalence of VUR is increased in selected groups, such as children with UTI, first-degree relatives of patients with VUR and children with antenatal hydronephrosis [74]. This was the reason for the development of recommendations for the investigation for VUR in these groups.

### Children with a first upper UTI

The guidelines of the American Academy of Pediatrics for all febrile children having their first UTI below 2 years of age include a combination of US and VCUG or RNC [75]. There is no benefit in delaying the performance of VCUG or RNC as long as the child is free of infection and bladder irritability is absent [75]. Swedish and United Kingdom guidelines are similar, but also include a DMSA [76, 77]. However, these guidelines do not reflect changing trends in the evaluation of children with UTIs. Recently, it has been suggested that a DMSA scan in these patients may replace VCUG as a first investigation, based on the fact that a normal DMSA scan excludes VUR of clinical significance [29]. VCUG is recommended only in patients with renal lesions on DMSA scan or recurrent febrile UTIs [28, 29]. It has also been suggested that DMSA should be performed within a few days after the diagnosis of APN, as the number of positive studies decreases rapidly following the initiation of antibiotic therapy [78].

It has to be pointed out that false-positive urine cultures are frequent and should be appropriately ruled out to protect children with a false diagnosis of UTI from being subjected to non-justified investigations. For this reason, the American Academy of Pediatrics recommended that if an infant or young child 2 months to 2 years of age with unexplained fever is assessed as being sufficiently ill to warrant immediate antimicrobial therapy, a urine specimen should be obtained by suprapubic aspiration or transurethral bladder catheterization; the diagnosis of UTI cannot be established by a culture of urine collected in a bag [75]. However, some children with clinical and laboratory findings of APN have negative urine cultures, because of inappropriate use of antibiotics. Levtchenko et al. have shown that APN can be diagnosed in these patients with the findings of DMSA [78].

It is fundamental that infections of the upper urinary tract should be distinguished from UTIs without parenchymal involvement. The diagnosis of APN in children with febrile UTIs on the basis of clinical and laboratory observations is unreliable. Therefore, an acute DMSA scan can be very useful in diagnosing APN and for the identification of patients at risk for subsequent renal scarring [20]. It is also feasible to differentiate the defects of APN and permanent renal scarring with an acute DMSA scan. APN is characterized by focal areas of diminished uptake with a normal renal contour. In contrast, permanent renal defects appear as focal or generalized areas of diminished radioisotope uptake with thinning or flattening of the cortex, and in other cases renal scars appear as classic discrete wedge-shaped parenchymal defects. This differentiation becomes more difficult in cases of APN with pre-existent renal scarring [20]. The major advantage of an acute DMSA is the identification of primary lesions and their differentiation from secondary lesions, but this is not always possible [20].

The use of DMSA for early investigation of young febrile children with their first UTI is not generally accepted. Recently, a questionnaire related to DMSA in children with UTI was submitted to 30 experts. Only 58% of the experts are systematically performing this examination during the acute phase of infection [79]. The major criticism is that the findings of an acute DMSA may not change the management of individual cases. A normally sized kidney on US with or without VUR has a very low risk for cumulative damage by UTIs resulting in CRI. Most children with APN will show abnormalities that may not have long-term implications. In addition, a DMSA has a more than negligible radiation load, especially when repeated several times. Some centers recommend a VCUG or RNC after a febrile UTI and recommend that DMSA should be performed 6 months after the last UTI in all patients with VUR, and only in those patients without VUR who are considered at higher risk for renal abnormality, i.e., with at least two febrile UTIs or one febrile UTI and delayed antibiotic treatment [17].

Guidelines for older children differ between centers and are not evidence based. US and DMSA are usually used for the investigation of these patients, and VCUG or preferably RNC is used only in children with abnormalities seen on DMSA or US, or those with antenatal hydronephrosis or a family history of VUR. It is important to advise the families to maintain a high suspicion for further UTI. Obviously all children with recurrent febrile UTIs should be investigated with a VCUG.

Poor compliance to the recommendations by the American Academy of Pediatrics for a first UTI in children was found in a retrospective cohort study using Washington State Medicaid data. Actually, less than 50% of children who were diagnosed with a UTI in their 1st year of life received either timely anatomic imaging or imaging for VUR [80]. Recently, the evidence for routine VCUG has been increasingly questioned, and possibly this was one of the reasons for the poor compliance in this study. In recent years there has been a tendency to neglect important data that has accumulated from the experience of many experts over the last 4 decades. However, the absence of evidence is not evidence of the absence.

In conclusion, there are two strategies for the initial investigation of children below 2 years of age with a first febrile UTI. The first recommends a DMSA scan and a US, and only children with renal lesions on DMSA scan or with recurrent febrile UTIs should be evaluated with a VCUG or RNC. It is usually feasible to distinguish primary and secondary renal lesions with this approach. This is the preferred procedure in centers with a legitimate academic interest. The alternative strategy adopted by many clinically oriented pediatric nephrologists is the use of a VCUG or RNC and a US for the initial investigation of these patients. A DMSA scan is recommended in a later stage only in patients with VUR or with recurrent UTIs.

### First-degree relatives of patients with VUR

The incidence of sibling VUR is significantly higher compared with the general population, and most studies advocate screening asymptomatic siblings of patients with VUR [81]. VUR was found in 32% of 570 siblings in a review from 11 publications [6]. However, VUR was greater than grade III in only 2%, and renal abnormalities were identified in 3% of siblings. These data do not prove that screening and treating asymptomatic siblings decrease infectious renal scarring [6]. In another study, a group of 117 asymptomatic siblings of known patients with VUR older than 5 years as well as younger children whose parents refused VCUG were screened with US. A VCUG was performed on children with US abnormalities (discrepancy in renal size, renal scarring or hydronephrosis, or a change in the size of the renal pelvis). Only nine patients had abnormal US, and VUR was diagnosed in five of them [82]. Obviously the incidence of VUR in the remaining 108 patients is not known, since the absence of abnormal findings in US does not exclude VUR. VCUG screening is probably unnecessary in siblings older than 5 years, since there is evidence that they have a lower incidence of VUR compared with younger children [83, 84].

Young asymptomatic siblings should be investigated with a VCUG or RNC. US might be a reliable alternative to invasive VCUG screening in older children. However, studies of control groups that consider sibling age are still needed to determine the benefit of screening asymptomatic siblings [6]. Families of these children should be advised to maintain a high suspicion for UTIs, and the treatment of diagnosed UTIs should start immediately.

### Children with antenatal hydronephrosis

The wider use of antenatal US resulted in an increased diagnosis of abnormalities of the urinary tract. Fetal renal pelvis dilatation is the most common abnormality, observed in 4.5% of pregnancies [85]. Significantly more dilating VUR was found in neonates with UTIs detected within the first 4 postnatal weeks compared with antenatally diagnosed patients (53% versus 29%). The incidence of congenital renal lesions was 14% in both groups. Focal renal scars developed during follow-up in 19% of renal units with VUR of grades IV and V, exclusively in the postnatal patient group [86]. Findings were similar in the study by Garin et al., who documented that only patients with grade IV-V VUR are at high risk for serious adverse outcome [87]. Infants with a history of fetal renal pelvis dilatation should have a postnatal US after the 1st week to avoid the false-negative results that occur in this period [88]. During the last decade, improved US technology has led to a significant increase of the antenatal identification of newborns with a small renal pelvis dilatation and the postnatal diagnosis of infants with low-grade VUR. In a recent study, a VCUG was performed only in infants with antenatal hydronephrosis and abnormal neonatal US findings. This policy resulted in decreasing the number of VCUG by 50% [5]. Using these restrictive recommendations, low-grade VUR was diagnosed in 74% of cases and a high rate of spontaneous resolution occurred at 24 months [5].

In conclusion, only infants with a history of fetal renal pelvis dilatation and postnatal hydronephrosis should be investigated with a VCUG or RNC. However, occasional cases of low-grade VUR could be missed with this approach, and all parents should be informed that if their child develops a fever of unknown origin, then the urine should be investigated for infection as soon as possible.

## Timing of follow-up in children with VUR

Since the majority of children will have resolution of VUR over time [89], most of them initially are treated with long-term antibiotic prophylaxis, and a periodic VCUG is performed until the spontaneous resolution of VUR [90]. However, no clear guidelines exist regarding the timing of follow-up by VCUGs. Recently, a follow-up with VCUG every 2 years in children with mild VUR and every 3 years in children with moderate/severe VUR has been suggested. With these recommendations, the number of VCUGs and the cost will be reduced significantly [91].

## Conclusions

Early identification of patients at risk for the development of acquired renal scarring (i.e., infants and young children after the first UTI or with antenatal diagnosis of hydronephrosis or siblings of patients with VUR) is the goal of the diagnostic imaging. This risk is significant if dilating VUR is not detected until the first UTI. Diagnostic delay, inappropriate treatment and dysfunctional voiding are important factors for the development of RN. However, the decline of renal function occurs mainly in patients with severe bilateral renal damage, usually with congenital RN, and UTIs have an important role in the deterioration of these lesions. Therefore, the focus of investigation should be based on what is happening to the kidney, and an early DMSA scan can identify those children who are at risk for pyelonephritic damage and subsequent renal scarring. With this approach, the number of children who need to be investigated with a VCUG will be reduced. However, this approach is not generally accepted, and the effect of early identification of renal damage on outcome is still unclear. Less invasive techniques with less radiation load should be used, and targeted imaging guidelines should be developed based on evidence from appropriate long-term studies. Our attention should not only focus on the strategies of imaging, but also on advising the parents of children at risk of developing permanent renal damage with simple preventive information.

Multiple choice questions (answers appear following reference list)

(More than one answer might be correct)

1. The prevalence of vesicoureteral reflux (VUR) is increased among:
  - Children with urinary tract infections (UTI)
  - Siblings with known VUR
  - Monozygotic twins
  - Neonates with antenatal diagnosis of hydronephrosis
  - Infants with UTI, compared to older children
2. Which of the following are true for renal scarring caused by UTI?
  - It is a risk factor for long-term complications such as hypertension and impaired renal function
  - It occurs only in children with VUR
  - DMSA scan is more sensitive than intravenous urography (IVU) to detect renal damage
  - Renal scars take months after an acute pyelonephritis to be detected with IVU.
  - Recurrent UTIs increase the risk for renal scarring
3. Which of the following are true of primary reflux nephropathy?
  - Boys are more often affected
  - The kidneys are normal at birth
  - Renal damage is the result of neonatal UTIs
  - VUR is usually of high grade
  - None of the above
4. Which of the following are indirect ultrasound signs, indicating VUR?
  - Pelvic dilatation
  - Thickening of the renal pelvic, ureteric or bladder wall
  - Residual volume after voiding
  - Increased renal size
  - None of the above
5. Febrile children with UTI below 2 years of age should be evaluated with:
  - An IVU and VCUG
  - A VCUG
  - An ultrasound
  - A DMSA scan
  - An ultrasound and DMSA scan

**Answers:**

Answer: a, b, d, e

Answer: a, c, d, e

Answer: a, d

Answer: a, b, c

Answer: e
